# Modeling the bound conformation of Pemphigus Vulgaris-associated peptides to MHC Class II DR and DQ Alleles

**DOI:** 10.1186/1745-7580-2-1

**Published:** 2006-01-21

**Authors:** Joo Chuan Tong, Jeff Bramson, Darja Kanduc, Selwyn Chow, Animesh A Sinha, Shoba Ranganathan

**Affiliations:** 1 Department of Biochemistry, The Yong Loo Lin School of Medicine, National University of Singapore, 8 Medical Drive, Singapore 117597; 2 Institute for Infocomm Research, 21 Heng Mui Keng Terrace, Singapore 119613; 3 Department of Dermatology, Weill Medical College of Cornell University, 525 East 68th Street, Rm. F-340, New York, NY 10021, USA; 4 Center for Investigative Dermatology, Division of Dermatology and Cutaneous Sciences, College of Human Medicine, Michigan State University, 4120 Biomedical and Physical Sciences Building, East Lansing, MI 48824, USA; 5 Department of Chemistry and Biomolecular Sciences & Biotechnology Research Institute, Macquarie University, NSW 2109, Australia

## Abstract

**Background:**

Pemphigus vulgaris (PV) is a severe autoimmune blistering disorder characterized by the presence of pathogenic autoantibodies directed against desmoglein-3 (Dsg3), involving specific DR4 and DR6 alleles in Caucasians and DQ5 allele in Asians. The development of sequence-based predictive algorithms to identify potential Dsg3 epitopes has encountered limited success due to the paucity of PV-associated allele-specific peptides as training data.

**Results:**

In this work we constructed atomic models of ten PV associated, non-associated and protective alleles. Nine previously identified stimulatory Dsg3 peptides, Dsg3 96–112, Dsg3 191–205, Dsg3 206–220, Dsg3 252–266, Dsg3 342–356, Dsg3 380–394, Dsg3 763–777, Dsg3 810–824 and Dsg3 963–977, were docked into the binding groove of each model to analyze the structural aspects of allele-specific binding.

**Conclusion:**

Our docking simulations are entirely consistent with functional data obtained from *in vitro *competitive binding assays and T cell proliferation studies in DR4 and DR6 PV patients. Our findings ascertain that DRB1*0402 plays a crucial role in the selection of specific self-peptides in DR4 PV. DRB1*0402 and DQB1*0503 do not necessarily share the same core residues, indicating that both alleles may have different binding specificities. In addition, our results lend credence to the hypothesis that the alleles DQB1*0201 and *0202 play a protective role by binding Dsg3 peptides with greater affinity than the susceptible alleles, allowing for efficient deletion of autoreactive T cells.

## Introduction

Major histocompatibility complex (MHC) class II molecules are heterodimeric glycoproteins consisting of α and β chains, with approximate molecular mass of 33 kDa and 28 kDa respectively. MHC class II molecules are specialized peptide receptors that play a critical role in initiating and regulating immune responses by binding peptide fragments that are 10–30 amino acids long [1] and present them on the surface of antigen-presenting cells for recognition by CD4^+ ^T cells. The class II region encodes genes for the human leukocyte antigen (HLA) or histocompatibility molecules class II structural genes DP, DQ and DR [2,3]. While specific DP, DQ or DR alleles at the HLA class II locus have been shown to correlate with particular autoimmune diseases, a variety of confounding factors including strong linkage disequilibrium between the different HLA alleles, especially DR and DQ, complicates the exact identification of MHC susceptibility alleles.

Pemphigus Vulgaris (PV) is a potentially life-threatening form of autoimmune blistering skin disorder due to loss of integrity of normal intercellular attachments within the epidermis and mucosal epithelium. The disease is characterized by the presence of pathogenic autoantibodies directed mainly against a 130-kDa transmembrane glycoprotein, desmoglein-3 (Dsg3) [4], within the desmosomes of the spinous layer of the skin. Strong association of PV to the major histocompatibility complex class II serotypes DR4 and DR6 have been reported in the literature [5-7] with over 95% of PV patients possessing one or both of these alleles [7]. Direct nucleotide sequence analysis of DR4 and DR6 subtypes revealed that susceptibility to PV is strongly linked to DRB1*0402 and DQB1*0503 molecular subtypes, respectively [7,8].

The use of computational techniques has been instrumental in advancing epitope-based vaccine research, with much work focusing on predicting the binding specificities of peptides to MHC molecules. Sequence-based predictive systems, based on identifying patterns in peptides with experimentally determined binding strength, are widely used to facilitate the identification of binding peptides to MHC class II molecules. Southwood *et al*. [9] developed a scoring matrix for DRB1*0401 based on a polynomial technique. Mallios [10,11] reported the results of an iterative stepwise discriminant analysis meta-algorithm to identify binders from non-binders for DRB1*0101. Brusic *et al*. [12] applied a genetic algorithm to discriminate binders from non-binders for DRB1*0401. Noguchi *et al*. [13,14] utilized both fuzzy neural network and hidden Markov model to predict potential binders to DRB1*0401 and DRB1*0101. Hammer *et al*. [15] employed a peptide side chain scanning technique for screening peptides that interact with DRB1*0401. Nielsen *et al*. [16] used a Gibbs sampling method for discriminating DRB1*0401 specific binders from non-binders. Karpenko *et al*. [17] made use of an ant colony system to search for DRB1*0401 binding and non-binding peptides. Doytchinova and Flower [18] employed an additive method for predicting the binding affinity of peptides bound to DRB1*0401, DRB1*0101 and DRB1*0701 based on the sum of the contributions of the amino acids at each position of the bound peptide and various interactions between them. However, despite recent advances in sequence-based predictive techniques, computational models for the majority of PV implicated alleles have been lacking, mainly due to the paucity of sufficient peptides as training data, and are unsuitable for predicting peptide binding to PV implicated alleles. Also, most computational methods focus on predicting just peptide binders and non-binders, whereas our aim is to distinguish between different modes of binding conferred by susceptible and protective alleles.

An alternative approach to predicting peptide/MHC (pMHC) complexes without the need of a large training dataset is to use information derived from three-dimensional structures. Logean and Rognan [19] utilized a combinatorial built-up algorithm to construct the three-dimensional structure of pMHC complexes. Altuvia *et al*. [20] reported the use of a computational threading approach to rank potentially binding peptides to MHC class I molecules. Lim *et al*. [21] employed molecular dynamic simulations to examine the structures of A*0201 in complex with 9-mer peptides. Michielin *et al*. [22,23] applied homology modeling to select peptides that bind to A*0201.

In addition to predicting the binding specificities of peptides to MHC molecules, three-dimensional models have also been used for structural classification of alleles into HLA "supertypes" based on structural features derived from the binding sites. Recently, Doytchinova *et al*. [24,25] employed hierarchical clustering and principal component analysis to classify alleles based on structural features into eight HLA class I and twelve HLA class II supertypes. The structural classification of alleles facilitates the identification of allelic subgroups that may share similar binding specificities and shed light into their possible role in cellular immunity against pathogens.

In the present study, we have attempted to understand the functional correlation between MHC class II alleles and PV, from a structural interaction view point. Molecular modeling of ten PV associated and non-associated MHC class II receptors (DR4: DRB1*0401, *0402, *0404, *0406, DR6 (also classified now as DR14): DRB1*1401, *1404, *1405, DQ2: DQB1*0201, *0202 and DQ5: DQB1*0503) were performed to explore the structural organization of the binding groove of these alleles. Nine previously identified epitopes, Dsg3 96–112, Dsg3 191–205, Dsg3 206–220, Dsg3 252–266, Dsg3 342–356, Dsg3 380–394, Dsg3 763–777, Dsg3 810–824 and Dsg3 963–977 (numbered in accordance with Swiss-Prot [26] accession number P32926), capable of stimulating patient derived T cells, were selected. The binding of these peptides to the DR and DQ structural models were studied by our efficient computational docking protocol [27]. In the light shed by these atomic models, the binding specificities of each allele to the various Dsg3 peptides are discussed. The results obtained in the study are able to discriminate between PV associated and non-associated alleles, consistent with the experimental results obtained by Veldman *et al*. [28] and Sinha *et al*. [unpublished results for Dsg3 342–356, 810–824 and 963–977]. Insights into structural features behind the immune response provided by protective alleles for PV have also been obtained by our structural immunoinformatics approach.

## Results and discussion

### Allele comparisons – HLA DR4 PV

**Figure 1:**
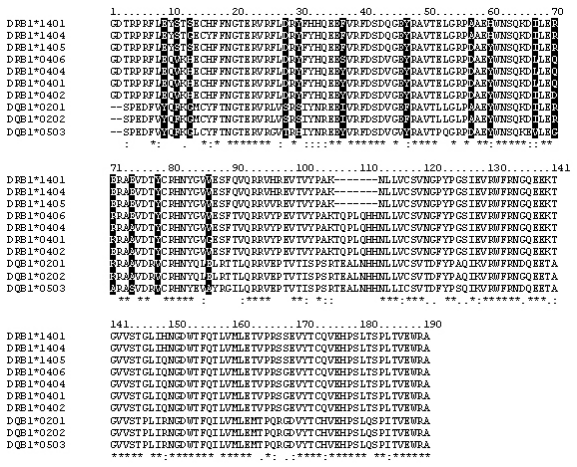
Multiple sequence alignment of the MHC DR and DQ alleles β chain. Pocket residues are shaded in black.

The sequence identity between the DR4 alleles (excluding DRB1*0401) with their corresponding templates ranges from 97.9 to 99.0%, and the sequence similarity (representing identical and conservatively substituted residues) was between 98.4 and 99.5% (Table 1). All five important peptide-binding pockets 1, 4, 6, 7 and 9 show extremely high structural conservation at the Cα positions, suggesting that any peptide discrimination leading to epitope selection between the alleles is mainly due to the size and nature of the side chains of the pocket residues. In order to further isolate the true disease-relevant allele within a haplotype, we compared specific residues in the polymorphic pockets regarded as important in conferring specificity for antigen presentation (Figure 1). Pocket 1, characterized by a Val/Gly β86 dimorphism, is the deepest cavity and thus, the most important anchor for peptide binding [29]. In addition, the functional specificity of DR4 molecules is also affected by polymorphisms at position β70, β71, β74, which contribute to pocket 4. Two negatively charged residues at position β70 and β71 that were previously suggested to influence peptide selectivity in PV patients [30] could be found in DRB1*0402 (Asp β70 and Glu β71) but a positively charge Arg/Lys β71 was found in DRB1*0404, *0406 and *0401. Amino acid polymorphism can also be observed at position β11 of pocket 6, β71 of pocket 7 and β37 of pocket 9 respectively.

**Table 1:** Sequence and structural similarity between the eight (DRB1*0402, *0404, *0406, *1401, *1404, *1405, DQB1*0202, and *0503) MHC structural models and their corresponding template structures (1D5Z: DRB1*0401, 1S9V: DQB1*0201, 1UVQ: DQB1*0602). Positives represent a measure of sequence similarity, accounting for identical and conservatively substituted residues. Root mean square deviations (RMSD) values in Å are shown for the Cα atoms of both MHC chains and for the residues comprising the different peptide-binding pockets.

**Allele**	**Template**	**Sequence Identity**	**Positives**	**Cα RMSD (Å)**					
				
				**α & β chains**	**Pockets**				
					
					**P1**	**P4**	**P6**	**P7**	**P9**
DRB1*0402	1D5Z	97.9%	99.0%	0.35	0.12	0.06	0.07	0.10	0.09
DRB1*0404	1D5Z	99.0%	99.5%	0.31	0.15	0.10	0.06	0.07	0.18
DRB1*0406	1D5Z	97.9%	98.4%	0.32	0.11	0.15	0.07	0.11	0.22
DRB1*1401	1D5Z	94.1%	97.3%	0.25	0.11	0.09	0.02	0.09	0.18
DRB1*1404	1D5Z	85.8%	89.5%	0.29	0.12	0.10	0.02	0.06	0.22
DRB1*1405	1D5Z	81.0%	83.2%	0.24	0.11	0.07	0.02	0.08	0.07
DQB1*0202	1S9V	98.0%	99.0%	0.57	0.16	0.09	0.04	0.15	0.05
DQB1*0503	1UVQ	93.0%	96.0%	0.39	0.03	0.07	0.01	0.10	0.06

### Allele comparisons – HLA DR6 PV

Study of individual allele frequencies in DR6 PV patients revealed that the relevant disease susceptibility allele is DQB1*0503 instead of DR6 alleles [Sinha *et al*., manuscript in preparation]. DQB1*0503 and the DR6 PV non-associated alleles investigated in this study show a significant degree of overlap in alignment, with 14 amino acid differences in areas of the binding cleft that could affect peptide binding. Clear differences in the amino acid sequences are observed at residue β86 of pocket 1, residues β13, β70, β71, β74, β78 of pocket 4, residue β11 of pocket 6, residues β28, β30, β67, β71 of pocket 7 and residues β9, β37, β57, β60 of pocket 9. Similar to the DR4 alleles, all five important peptide-binding pockets 1, 4, 6, 7 and 9 in DBQ1*0503 and DR6 alleles demonstrate exceptionally high structural conservation at the Cα positions. A significant difference is that DQB1*0503 contains a negatively charged Asp β57 that differs from the uncharged Ala β57 found in non-PV associated DRB1*1401 and *1404. Also, at positions β70 and β71, DQB1*0503 does not contain negatively charged residues identified in DRB1*0402 that are critical for binding of self-antigens in DR4 PV patients. Instead, these positions were replaced by two small neutral hydrophobic residues (Gly β70 and Ala β71), suggesting that DRB1*0402 and DQB1*0503 may recognize different sets of PV epitopes under the influence of a different balance of intermolecular forces. Positions β70 and β74 show charge reversal, in the non-PV associated DRB1*1401, *1404 and *1405 alleles while the negative charge at β71 alone is conserved, compared to DRB1*0402, making pocket 4 the single dominant factor discriminating between PV non-association and susceptibility.

### Allele comparisons – PV protective and susceptible alleles

Differences in the amino acid sequences are observed at residue β86 of pocket 1, residue β70, β71 of pocket 4, residues β28, β30, β47, β71 of pocket 7 and residues β37, β57 of pocket 9. Both protective alleles (DQB1*0201 and DQB1*0202) do not contain negatively charged residues at position β70 (pocket 4) and β71 (pocket 7). Instead, these positions were replaced by two large and positively charged amino acids (Arg β70 and Lys β71). The functional specificities of PV protective and susceptible alleles are also affected by clear structural differences in the Cα positions of both α and β chains (Cα RMSD > 0.57Å) indicating that any differences in peptide discrimination between the alleles is due to a combination of both the backbone conformation as well as the size and nature of the side chains of the pocket residues.

### Epitope comparisons – HLA DR4 PV

Eight previously identified stimulatory Dsg3 epitopes (Dsg3 191–205, Dsg3 206–220, Dsg3 252–266, Dsg3 342–356, Dsg3 380–394, Dsg3 763–777, Dsg3 810–824 and Dsg3 963–977) for DRB1*0402 were docked into the binding groove of all DR4 (DRB1*0401, *0402, *0404, *0406) alleles investigated in this study. Analysis of these Dsg3 peptide-bound alleles revealed that only one peptide conformation can fit perfectly into the binding cleft of DRB1*0402, and atomic clashes of these Dsg3 peptides are obtained for all other DR4 subtypes investigated in this study. Notably, two epitopes (Dsg3 342–356 and Dsg3 810–824) have small residues (Ser/Cys) in pocket 1, suggesting that large anchor residues may play a critical role for high affinity binding in DR4 PV molecules, an observation previously documented for influenza-associated I-A^d ^allele of mice [31]. This finding provides support to the evidence that DRB1*0402 is associated with PV whereas other DR4 subtypes are non-associated, with the exception of DRB1*0406 that is reported to be associated in the Japanese population [32]. As such, there is a possibility of the existence of other peptides relevant in the Japanese populations that bind to *0406 but are yet to be determined.

### Epitope comparisons – HLA DR6 PV

Dsg3 96–112, a recently identified epitope in DR6 PV patients [28], fits perfectly into the binding groove of DQB1*0503 with two identified core sequences at residues 101–109 and residues 102–110. The identified 101–109 core has four intermolecular hydrogen bonds compared to seven intermolecular hydrogen bonds in the core of 102–110. Perfect fitting of Dsg3 206–220, Dsg3 252–266, Dsg3 342–356, Dsg3 810–824 and Dsg3 963–977 into the binding groove of DQB1*0503 is also obtained. Atomic clashes are obtained for Dsg3 191–205, Dsg3 380–394 and Dsg3 763–777 as well as all DR6 alleles investigated in this study. The proportion of DRB1*1401, *1404 and *1405 has been reported to be increased in PV probably due to linkage disequilibrium. The lack of binding of all stimulatory peptides investigated in this study to these alleles indicates that the HLA association in DR6 PV patients is more likely at the DQB1 locus (DQB1*0503 allele) and not the linked DRB1 loci (DRB1*1401, *1404 and *1405). Our data supports the notion that the reported associations of this disease with DRB1*1401, *1404, *1405 are due to linkage disequilibrium with the true disease associated allele (DQB1*0503).

### Epitope comparisons – PV susceptibility Alleles

Our docking simulations reveal strong evidence that DRB1*0402 and DQB1*0503 can bind to different sets of PV epitopes by recognizing different core peptide sequences in the binding groove (Table 2). Three PV epitopes (Dsg3 191–205, Dsg3 380–394 and Dsg3 763–77) can only bind to DRB1*0402, four PV epitopes (Dsg3 206–220, Dsg3 252–266, Dsg3 342–356 and Dsg3 810–824) can bind to both alleles with different core peptide sequences, one PV epitope (Dsg3 963–977) can bind to both alleles with the same core peptide sequence, and one PV epitope (Dsg3 96–112) can only bind to DQB1*0503. DRB1*0402 and DQB1*0503 may recognize the same Dsg3 epitope at two unique sets of core sequences (which may be in close proximity) within the epitope itself. These findings are completely in accord with experimental data [28].

**Table 2:** Preferred core residues for PV associated alleles. Best fitting nonameric core residues in the binding groove are underlined.

**No.**	**Residues**	**Allele**	**Core peptide sequences**	**No.**	**Residues**	**Allele**	**Core peptide sequences**
I	96–112	DRB1*0402	-	V	342–356	DRB1*0402	SVKLSIAVKNKAEFH
		DQB1*0503	PFGIFVVDKNTGDINIT			DQB1*0503	SVKLSIAVKNKAEFH
			PFGIFVVDKNTGDINIT	VI	380–394	DRB1*0402	GIAFRPASKTFTVQK
II	191–205	DRB1*0402	NSKIAFKIVSQEPAG			DQB1*0503	---
		DQB1*0503	---	VII	763–777	DRB1*0402	SGTMRTRHSTGGTNK
III	206–220	DRB1*0402	TPMFLLSRNTGEVRT			DQB1*0503	---
		DQB1*0503	TPMFLLSRNTGEVRT	VIII	810–824	DRB1*0402	NDCLLIYDNEGADAT
IV	252–266	DRB1*0402	ECNIKVKDVNDNFPM			DQB1*0503	NDCLLIYDNEGADAT
		DQB1*0503	ECNIKVKDVNDNFPM	IX	963–977	DRB1*0402	ERVICPISSVPGNLA
			ECNIKVKDVNDNFPM			DQB1*0503	ERVICPISSVPGNLA

### Epitope comparisons – PV protective alleles

Our simulation results indicate that DQB1*0201 and DQB1*0202 can bind to multiple core sequences for the majority of PV epitopes investigated in this study. DQB1*0201 can bind one epitope (Dsg3 963–977) at two core regions, one epitope (Dsg3 206–220) at three core regions, three epitopes (Dsg3 191–205, 252–266 and 342–356) at four core regions, and two epitopes (Dsg3 96–112 and 810–824) at five core regions. DQB1*0202 can bind two epitopes (Dsg3 96–112 and 963–977) at three core regions, two epitopes (Dsg3 342–356 and 810–824) at four core regions and one epitope (Dsg3 252–266) at five core regions. In contrast, the majority of PV epitopes (with the exception of Dsg3 96–112 and 252–266) can bind to PV susceptible alleles DRB1*0402 and DQB1*0503 at a single core. This finding lends support to the hypothesis that the protective alleles DQB1*0201, *0202 may be capable of binding to most peptides with greater affinity than PV susceptible alleles, allowing for efficient deletion of autoreactive T cells [33].

### Role of flanking residues in peptide selection

Our data demonstrates that the conformations of flanking peptide residues that extend beyond the binding groove are critical to peptide selection in MHC class II alleles. The core sequences of Dsg3 963–977 fit perfectly within the binding grooves of non-associated alleles DRB1*0401, *0404, and *1404 but poor contacts to the respective alleles at Phe α50 are obtained when the conformation of the N-terminal flanking residue Ile4 is taken into account. These results suggest that binding is determined by both the core and flanking segments while considering the overall interactions between each peptide and the respective alleles.

### Sequence motifs

Sequence-based epitope prediction relies on the identification of sequence motifs from available experimental data. The correlation of core peptide residues with binding motifs previously defined by Veldman *et al*. [28] and Sinha *et al*. (unpublished results) is shown in Table 3, to understand to what extent sequence-based approaches will be valid with specific reference to PV. The sequence conservation observed here is too low to warrant the generation of a consensus sequence pattern.

**Table 3:** Comparison of core peptides (numbering according to Table 2) from structural docking in the different binding pockets with the sequence-based binding motifs. '+' indicates compliance of amino acid residues within the core (bold underlined) with the respective binding motifs defined by the groups of ^a ^Veldman [28] and ^b^Sinha [2, unpublished results].

**No.**	**Residues**	**Peptide Sequence and positions in the** bound conformation for DRB1*0402			**Core peptide residue positions** ** as defined by binding motifs**							
			
					**p1**		**p4**		**p6**		**p9**	
			
			**1 2 3 4 5 6 7 8 9**		**V^a^**	**S^b^**	**V**	**S**	**V**	**S**	**V**	**S**
II	191–205	NSKIA	**F K I V S Q E P A**	G	+			+		+		+
III	206–220	TPM	**F L L S R N T G E**	VRT	+				+	+		+
IV	252–266	ECNI	**K V K D V N D N F**	PM					+	+		
V	342–356	SVKL	**S I A V K N K A E**	FH				+	+	+		+
VI	380–394	GIA	**F R P A S K T F T**	VQK	+			+		+		+
VII	763–777	SGT	**M R T R H S T G G**	TNK	+	+	+	+	+	+		+
VIII	810–824	ND	**C L L I Y D N E G**	ADAT				+		+		+
IX	963–977	ERVICP	**I S S V P G N L A**		+	+		+				+

Peptide VII (Dsg3 763–777) agrees well with the motifs from Veldman *et al*. [28] and Sinha *et al*. (unpublished results), while all other peptides show low to moderate compliance. Of the four positions compared, peptide IV (Dsg3 252–266) shows agreement only at position p6. For Dsg3 342–356 peptide, the core nonamer identified by our models is 346–354, which is register-shifted by one residue from the core of 347–355 reported by Veldman *et al*. [28], and 345–353 identified by Sinha *et al*. (unpublished results), for the binding groove of *0402. This shift is critical as residues p1 and p4 identified by us do not fit well into both binding motifs. Our modeling studies suggest that peptide position p4 need not be positively charged as indicated by Veldman *et al*. [28], supporting the existence of a more degenerate motif by Sinha *et al*. (unpublished results) at this position. In addition, p1 also appears to be more degenerate than previously suggested [28], showing a preference for hydrophobic and large residues but can accommodate residues of other sizes as well. Hence for generating sequence patterns to design peptides for vaccine design, structural information is important [34] and the exact peptide in the binding groove identified by our docking protocol will be most useful here.

### Disease progression in PV

T cell response to a number of epitopes among PV patients has been reported in several studies [2,8,26,29,30]. There may be disease heterogeneity, meaning that clinically similar but distinct phenotypes could operate by alternate pathways, each with a different initial immunodominant epitope(s). The differential T cell reactivities among individual patients to individual peptides may also be a function of the disease stage or severity and correlate with mechanisms of disease progression. While there may be a limited set of epitopes present in patients in the early stages of the disease, epitope spreading can occur during disease progression, resulting in reactivity to previously innocuous epitopes. In addition, reactivities to multiple epitopes within individual patients were detected in two cases (Dsg3 191–205 and 342–356 for PV107; Dsg3 191–205, 810–824 and 963–977 for PV117). Autoantibodies against desmoglein 1 have also been reported in severe disease [35]. One other incidence of multiple T cell reactivities within a PV patient has been previously reported [29]. These findings, together with our simulation results, lend further credence to the hypothesis that no single epitope is responsible for both disease initiation and propagation and are consistent with the expected and observed ability to generate multiple pMHC complexes from a single target autoantigen.

## Conclusion

Docking simulations at the binding site of PV associated and non-associated DR and DQ alleles have been performed to analyze the structural aspects of binding and allele-specificity for nine previously identified Dsg3 epitopes. To represent the possibility that any core peptide sequences can be recognized by the binding groove of MHC class II alleles, a sliding window was applied to generate all possible combinations of core nonamer peptides from each Dsg3 peptide. This method can eliminate any bias in selecting core peptides based on sequence patterns alone.

We have found the existence of best-fit core residues at different positions of each peptide (excepting Dsg3 96–112) into the binding groove of DRB1*0402 with no observed atomic clash penalties or bad contacts. In contrast, atomic clashes are experienced in all other PV non-associated DR4 alleles. This discrimination further establishes the crucial role that DRB1*0402 plays in selecting specific self-peptides in DR4 PV. In addition, we found that DRB1*0402 and DQB1*0503 do not necessarily share the same core residues. It is possible that DRB1*0402, DQB1*0503 and all other PV non-associated alleles may have different sets of binding specificities. Our studies also indicate that perfect fitting of the core nonameric peptide residues within the binding groove of MHC class II alleles may not guarantee perfect fitting of the entire peptide, and flanking residues outside the binding groove may play a critical part in peptide selection. Such binding interactions suggest that longer peptides extending out of the binding groove of MHC class II alleles must be taken into account in the generation of HLA class II binding motifs and for vaccine design.

The comparison of core peptide residues with binding motifs previously defined by Veldman *et al*. [28] and Sinha *et al*. (unpublished results) indicates that sequence-based methods are currently insufficient for the design of PV epitopes as there are both register shifts in the suggested motifs as well as polymorphism observed in the core residues in the binding groove. More experimental data are necessary for the definition of DR4 and DR6 PV specific binding motifs.

The methodology presented here may serve as a general method suitable for finding allelic specific peptides, applicable to the design of both sub-type specific vaccines as well as promiscuous peptide epitopes. In particular, this approach is useful in situations where there is insufficient data for training sequence-based predictive models. In the context of PV, this approach provides a means for discriminating between peptide binders and non-binders for a number of PV implicated alleles where training data is deficient. Our results support the hypothesis [33] that the alleles DQB1*0201 and *0202 play a protective role by binding Dsg3 peptides with greater affinity than the susceptible alleles, facilitating efficient deletion of autoreactive T cells. With increasing evidence indicating that no single epitope may be responsible for both disease initiation and propagation in PV, it is valuable to identify all Dsg3 peptides that bind to the PV susceptible alleles. Specifically, the identification of peptides that bind to both DRB1*0402 and DQB1*0503 are of great importance as these peptides may serve as targets for epitope-based therapeutic vaccination of both DR4 and DR6 PV patients. Future work will include autoantigens from Dsg1, the main causative agent for pemphigus foliaceus and reported in severe cases of PV.

## Materials and methods

### Template search

In this study, ten PV associated, closely related non-associated and protective MHC class II alleles DRB1*0401, *0402, *0404, *0406, *1401, *1404, *1405, DQB1*0201, *0202, and *0503 were selected for analysis. MHC sequence data were obtained from IMGT-HLA [36] database. The α chain of all DR alleles investigated in this study is the DRA1*0101 sequence, with the β chain from the allele sub-type. To identify potential structural templates available in the Protein Data Bank (PDB) [37] for model building, a sequence similarity search was performed using PSI-BLAST [38] running on the servers at NCBI  and the highest quality templates were selected among the returned results. Among these, the crystal structures of HLA-DR4 (PDB code 1D5Z) and HLA-DQ2 (PDB code 1S9V) were adopted as the structures of DRB1*0401 and DQB1*0201 respectively (100% sequence identity). The crystal structures of DRB1*0401 (PDB code 1D5Z), DQB1*0602 (PDB code 1UVQ) and DQB1*0201 (PDB code 1S9V) were selected as templates for all other DR subtypes, DQB1*0503 and DQB1*0202 respectively (Table 1).

### Model building

The program MODELLER [39] was employed for comparative modeling of both DRB1 (*0402, *0404, *0406, *1401, *1404, *1405) and DQB1 (*0202, *0503) subtypes. The models are constructed by optimally satisfying spatial constraints obtained from the alignment of the template structure with the target sequence and from the CHARMM-22 force field [40]. The initial model was refined by assigning the rotameric states of essential side chains according to the corresponding crystal structure, followed by a short energy minimization [41] from the program Internal Coordinates Mechanics (ICM; Molsoft LLC, San Diego, CA) [42].

### Patient recruitment and groupings

Patients and controls were recruited from the Dermatology clinics at New York Presbyterian Hospital (Cornell Campus, New York, NY). HLA typing was performed at the Rogosin Institute, New York Presbyterian Hospital, NY. Controls were without disease and were HLA types DRB1*0402 (*n *= 1), or DRB1*0402 and DQB1*0503 (*n *= 1); while PV patients typed as DRB1*0402 (7/9), DRB1*0402 and DQB1*0503 (1/9), or DQB1*0503 (1/9) (Table 4).

**Table 4:** HLA class II haplotypes of PV patients (PV) and controls (CR). Typing was performed at the Rogosin Institute, New York Presbyterian Hospital, NY.

**Subject**	**Sex**	**Age**	**DRB1**	**DQB1**	**DQA**
PV104	F	64	*0402, *0403	*0302, *0304	*03011
PV105	F	73	*1404, *0102	*0503, *0501	*01041, *0101
PV107	F	70	*0402, *0102	*0302, *0501	*03011, *0101
PV108	F	52	*0402, *0404	*0302, *0304	*03011, *0303
PV112	F	57	*0402, *1101	*0302, *0301	*03011, *0505
PV114	M	50	*0402, *1401	*0503, *0302	*03011, *01041
PV115	F	34	*0402, *0701	*0302, *0202	*03011, *0201
PV117	M	44	*0402, *0403	*0302, *0305	*03011
PV118	M	62	*0402, *1302	*0601, *0402	*0102, *0303
CR101	F	58	*0402, *0701	*0202, *0302	*0201, *0301
CR102	M	81	*0402, *1101	*0301, *0302	*0501-05, *0301

### Peptide set

Nine previously identified epitopes Dsg3 96–112, 191–205, Dsg3 206–220, Dsg3 252–266, Dsg3 342–356, Dsg3 380–394, Dsg3 763–777, Dsg3 810–824 and Dsg3 963–977 that elicited primary proliferative T cell response in PV patients [2,8,26,29,30] were selected for modeling studies. T cell response to eight of these peptides (Dsg3 191–205, Dsg3 206–220, Dsg3 252–266, Dsg3 342–356, Dsg3 380–394, Dsg3 763–777, Dsg3 810–824 and Dsg3 963–977) has been reported in patients carrying DRB1*0402. Dsg3 96–112 has been reported to elicit T cell response in patients with DQB1*0503 but lacking DRB1*0402 [28]. Of these Dsg3 191–205, Dsg3 342–356, Dsg3 810–824 and Dsg3 963–977 were shown to directly bind to the DRB1*0402 receptor by competitive binding assays (Sinha *et al*., unpublished results). Briefly, soluble HLA DRA1*0101/DRB1*0402 were purified by DR-specific affinity chromatography and incubated with different concentrations of experimental peptides (0–40 μM) in the presence of biotinylated class II-associated invariant-chain peptide (CLIP) (1 μM) for 2 hours. The MHC-peptide complexes were then captured on a 96-well plate coated with anti-HLA-DR (L243) (BD Pharmingen, San Diego, CA). The CLIP bound to the MHC molecules was directly assayed using Europium (Eu)-labeled streptavidin (Perkin Elmer, Boston, MA). The relative binding of peptides was subsequently determined by measuring the displacement of the CLIP at different peptide concentrations.

### Peptide docking

**Figure 2:**
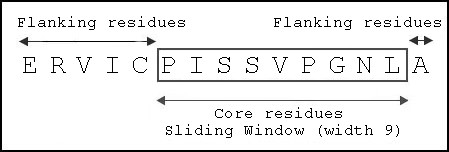
Sliding window of width 9 applied to identify core residues of Dsg3 963–977 to be modeled into binding groove.

Analysis of binding motifs [43] and available crystal structures suggested a core region of nine amino acids as essential for binding. Based on this observation, a sliding window input of size nine (Figure 2) is applied to generate all combinations of nonameric core peptide residues to be modeled into the binding groove of each allele. Each core peptide fragment is docked into the binding groove in a three-step protocol described in an earlier study [27]. In brief, docking of core peptide residues is performed as follows: (i) core peptide fragments at the ends of the binding groove is docked using ICM biased Monte Carlo procedure, followed by (ii) loop closure of peptide core residues by satisfaction of spatial constraints, and finally (iii) refinement of the backbone and side-chains of peptide core residues as well as atomic clash regions at receptor. Next, flanking residues are extended from the core peptide residues using ICM biased Monte Carlo procedure. The adopted empirical scoring function [44,45] takes into account continuum and discrete electrostatics, and hydrophobic and entropy loss [45,46]. This methodology allows rapid and accurate docking of peptides with an average computing time of approximately 18 minutes for the complete modeling of each peptide on a 4-CPU SGI Origin 3200 workstation. For each ligand, the best solution is obtained based on the following criteria: pattern of hydrogen bonding to the MHC molecule, pattern of hydrophobic burial of peptide side chains, and the absence of atomic clashes or repulsive contacts.

### Definition of contact residues

In this study, MHC-peptide residues were considered to be in contact if at least one pair of their non-hydrogen ("heavy") atoms was found to be within 4.00Å radius [47]. Intra-peptide interactions and intra-MHC interactions were not considered as they have minor influence on peptide/protein backbone structure. Any atom in the peptide and any atom in the MHC were considered to be experiencing atomic clash if their separation is below 2.00Å [48] for non-hydrogen atoms and below 1.60Å for atoms participating in hydrogen bonds [49,50].

### Definition of binding pocket for MHC Class II alleles

Interactions between side-chains of bound peptide ligands and polymorphic cavities (or anchor "pockets") in the binding site of MHC class II alleles are important in determining the peptide binding affinity and sequence specificity of MHC molecules and are defined according to the work of Stern *et al*. [51,52].

## List of abbreviations

PV: Pemphigus vulgaris

Dsg3: Desmoglein-3

MHC: Major histocompatibility complex

HLA: Human leukocyte antigen

## Authors' contributions

JCT carried out the homology modeling and docking studies and drafted the manuscript. JB, DK and SC carried out the immunoassays. AAS and SR participated in the design of the study. SR developed the project and finalized the manuscript.
